# C3 Hypocomplementemia Predicts the Progression of CKD towards End-Stage Kidney Disease in IgA Nephropathy, Irrespective of Histological Evidence of Thrombotic Microangiopathy

**DOI:** 10.3390/jcm13092594

**Published:** 2024-04-28

**Authors:** Giovanni Maria Rossi, Federico Ricco, Isabella Pisani, Marco Delsante, Umberto Maggiore, Enrico Fiaccadori, Lucio Manenti

**Affiliations:** 1 Nephrology Unit, University Hospital of Parma, 43126 Parma, Italy; giovannimaria.rossi@ao.pr.it (G.M.R.); federiccco1996@gmail.com (F.R.); isabellapisani88@gmail.com (I.P.); mdelsante@ao.pr.it (M.D.); umberto.maggiore@ao.pr.it (U.M.); enrico.fiaccadori@unipr.it (E.F.); 2 Dipartimento di Medicina e Chirurgia, Università di Parma, 43126 Parma, Italy; 3 Nephrology Unit, Azienda Sociosanitaria Liguria 5, 19121 La Spezia, Italy

**Keywords:** IgA nephropathy, complement, thrombotic microangiopathy, end-stage kidney disease

## Abstract

**Background:** IgA nephropathy (IgAN) is the most common primary glomerulonephritis worldwide. IgAN causes end-stage kidney disease (ESKD) in 30–40% of all cases. The activation of the complement system by pathological circulating IgAs, which is often associated with low serum C3 levels (LowC3), seems to play a crucial role. Previous studies have shown an association between histological evidence of TMA, which is the result of alternative complement activation, and poor outcomes. However, it is not known to what extent the decrease in serum C3 levels reflects ongoing TMA injury. Our study aimed at assessing the association between LowC3 and ESKD and whether this association reflects ongoing TMA. **Methods:** We enrolled all patients with biopsy-proven IgAN and followed-up patients until their last visit, ESKD, or death. **Results:** Of the 56 patients included in the study, 12 (21%) presented low serum C3 (LowC3) at the time of renal biopsy. TMA was significantly more frequent in the LowC3 group [7/12 (58%) vs. 9/44 (20%), *p* = 0.02]. After adjusting for potential confounders, LowC3 was strongly associated with an increased hazard of ESKD (hazard ratio [HR]: 5.84 [95%CI: 1.69, 20.15; *p* = 0.005). The association was not affected by adjusting for TMA. The estimated overall proportion of the relation between C3 and ESKD mediated by TMA was low and not statistically significant. **Conclusions:** Our study provides evidence that C3 hypocomplementemia is associated with an increased risk of ESKD through mechanisms that are largely independent from TMA.

## 1. Introduction

IgA nephropathy (IgAN), also known as Berger’s disease [1], is the most common primary glomerulonephritis worldwide [2]. Although benign or indolent in many cases, IgAN causes end-stage kidney disease (ESKD) in 30–40% of all cases within 20–30 years after diagnosis despite current treatment strategies [2,3,4] and reduces life expectancy by 10 years [5].

Considering the geographical differences in the epidemiology of IgAN between Europe and Asia, our definition of the disease may encompass different entities with different distributions [6]. Compared to white Europeans (31 cases per million population/y in France), Asian populations have a higher rate of IgAN (45 cases per million population/y). Men are twice as likely to be affected by IgAN in North America and Europe [7], while females are equally affected in Asia. In terms of ethnicity, the Asian population is much more likely to be affected by IgAN than the white population, which is, in turn, much more likely to be affected by IgAN than the black population. This disease most often presents between the teenage years and the late 30’s but can present at any age [8]. Within Europe, a more subtle south-to-north prevalence gradient has also been described; Northern Europeans have a 2.4-fold increased risk of ESRD from IgAN compared to Southern Europeans.

Diagnosis is based on immunofluorescence or immunohistochemical microscopic evidence of IgA-dominant or codominant mesangial immune deposits [1]. C3 deposits are identified in 90% of IgAN biopsies [9]. The activation of the complement system by pathological circulating IgAs (galactose-deficient IgA, GDIgA1) seems to play a crucial role. The current understanding of IgAN pathophysiology is based on a “multiple hit” hypothesis. IgAN patients produce higher amounts of Gd-IgA1 than healthy individuals, resulting in high circulating levels (first hit). The Gd-IgA1 molecules are recognized by IgG autoantibodies (second hit), forming immune complexes in the blood (third hit). The accumulation of immune complexes in the mesangium leads to the activation of mesangial cells and therefore inflammation, eventually causing kidney injury (fourth hit) [10]. However, the precise pathophysiology of IgA–mediated complement activation remains poorly understood. A variety of mechanisms are thought to be at play, which may act alone or in combination, i.e., (i) the stabilization of the C3 convertase, (ii) the activation of the lectin pathway through polymeric IgAs, and (iii) inherited partial deficiencies of complement regulatory proteins due to pathogenic gene variants [11].

Other than non-specific prognostic markers of progressive renal disease such as proteinuria, there is evidence that remission of hematuria represents a favorable prognostic marker [12,13].

Recently, the overactivation of the alternative complement pathway (ACP) has been recognized as a key driver of ESKD progression by means of progressive arteriolar damage and thrombotic microangiopathy (TMA) [14,15]. TMA refers to the histological evidence of small vessel thrombosis, in most cases involving the kidneys with organ damage, leading to acute or chronic kidney injury, with or without hematological signs (i.e., non-hemolytic microangiopathic anemia [MAHA] and thrombocytopenia), due to a variety of conditions and drugs [16]. In 2012, El Karoui showed that 53% of IgAN cases presented chronic or acute TMA [17], although the incidence of TMA in other studies was lower (2–40% of cases) [15,18,19,20,21]. One-third of TMA cases were associated with a picture of atypical hemolytic uremic syndrome (aHUS), i.e., non-immune microangiopathic hemolytic anemia (MAHA), thrombocytopenia, and acute kidney injury (AKI). Renal prognosis was significantly worse in IgAN cases with TMA, and the presence of aHUS further increased the risk of ESKD [14]. The reported discrepancies in terms of TMA incidence and presentation across studies might reflect the adoption of different histological definitions and differences in terms of distribution of genetic predisposition to aHUS, as well as the presence or lack of laboratory signs of TMA (i.e., hematological manifestations of TMA).

A recent study identified low serum C3 and a low C3/C4 plasma ratio as negative prognostic factors in IgAN [22]. Moreover, a low C3/C4 ratio at the time of kidney biopsy correlated with worse clinical and histological findings [23]. Another study, which did not assess the renal outcomes of hypocomplementemic patients, showed the negative prognostic role of microangiopathic lesions per se, which were associated with a higher incidence of hypocomplementemia; glomerular C3 staining intensity was also correlated with worse renal prognosis, the histological finding of TMA, and the degree of interstitial fibrosis [19]. In sum, these data suggest that progressive microangiopathic damage caused by ACP overactivation might be a significant determinant of disease progression in IgAN. Such progressive damage would be in line with the wide spectrum of disease severity typical of IgAN, ranging from indolent to rapid progression towards ESKD.

Based on these assumptions, our study aimed at assessing whether, in patients with IgAN, low serum C3 (LowC3) and the histological evidence of TMA, which are considered indirect signs of ACP overactivation, are associated with an increased risk of ESKD and whether the relationship between LowC3 and ESKD reflects the development of TMA. 

## 2. Materials and Methods

**Study population**. We enrolled all patients with biopsy-proven IgAN, followed from 1 April 2008 to 31 March 2019 by the Nephrology Unit of Parma University Hospital (Parma, Italy). The study was approved by the local Ethical Committee and all studied participants provided informed consent (protocol code 945/21-08-2019).

**Data collection**. We retrospectively followed-up patients until their last visit before 30 November 2023 or ESKD or death. We extracted clinical data at the time of renal biopsy and during follow-up from electronic medical records. The estimated glomerular filtration rate (eGFR) was calculated using the Chronic Kidney Disease Epidemiology Collaboration (CKD-EPI 2009) equation [24]. Serum complements C3 and C4 were measured with a nephelometric method that remained unchanged for the whole study period. 

**Histological diagnoses**. Renal biopsy specimens were routinely assessed by light microscopy and direct immunofluorescence. For light microscopy, formalin-fixed paraffin-embedded sections were stained with methenamine silver, periodic acid-Schiff (PAS), haematoxylin and eosin (H&E), and Masson’s trichrome. For direct immunofluorescence, the specimens were embedded in OCT (Tissue Tek, Miles Inc., Elkhart, IN, USA) and stored in liquid nitrogen. Samples were cut into 3 µm sections by a cryostat (Leica CM1850, Leica Mycrosystems, Wetzlar, Germany), placed on poly-L-lysine-coated glass, and stored at −80 °C until use. These sections were rinsed in 0.01 mol/L phosphate-buffered saline (PBS), pH 7.4, and incubated for 30 min at room temperature with fluorescein isothiocyanate (FITC)-conjugated rabbit antihuman IgG, IgA, IgM, kappa, lambda, C3c, C1q, and fibrinogen antisera (Dako, Copenhagen, Denmark). Negative controls were processed in parallel using PBS or an equivalent concentration of non-immune rabbit or mouse serum as primary antibodies. Samples were observed using a Leitz Diaplan microscope (Leica, Milan, Italy). The deposition of each antiserum was scored as “negative” (diffuse negative, focal slightly positive), “+” (diffuse slightly positive, focal moderately positive), “++” (diffuse moderately positive, focal intensely positive), and “+++” (diffuse intensely positive). All slides were reviewed by two independent expert renal pathologists (GMR, MD), who were blinded to the patients’ clinical data, and scored using the Oxford Classification of IgAN [25]. The presence of acute TMA (glomerular thrombi, endothelial edema, mesangiolysis, presence of fragmented red blood cells, arteriolar thrombi, arteriolar intimal edema, necrosis of myocytes, and/or fibrinoid necrosis) or chronic TMA (double contours of the capillary loops and/or hyperplastic arteriolosclerosis, i.e., “onion skin” appearance of arterioles) was assessed, and scored as either present or absent, chronic, or acute.

**Treatments.** Antiproteinuric therapy with RAAS was always introduced where possible, associating it with the use of steroids when proteinuria was greater than 1 g. In selected cases, therapy with other immunosuppressants was introduced. These criteria were consistent during the study period.

**Data analysis**. We performed all analyses using Stata 18 (StataCorp, College station, TX, USA). We regarded two-sided *p* values < 0.05 as statistically significant. We tested differences between two groups using Mann–Whitney test for continuous variables and Fisher’s exact test for categorical variables. We estimated ESKD-free crude survival using Kaplan–Meier estimators and tested the crude difference with the log-rank test. The time started from the time of diagnosis until the ESKD or last follow-up visit. No patient died or was lost to follow-up. We computed adjusted hazard ratios using multivariable Cox proportional hazards regression models. We adjusted the models for confounders that we selected based on background knowledge. We checked the linearity of continuous variables with fractional polynomials, and proportional-hazards assumptions using Schoenfeld residuals. We fitted the crude and adjusted Cox proportional hazards regression models with either TMA or a low C3 (separately), but also models that included both TMA and a low C3 in order to estimate the association with ESKD that was independent of either variable. In addition, using mediation analysis, we estimated the overall proportion mediated by TMA of the adjusted relation between a low C3 and ESKD. For this purpose, we used the Stata program med4way [26]. The program med4way estimates the components of the 4-way decomposition of the total effect of a low C3 on ESKD in the presence of the mediator TMA with which a low C3 may interact: the total effect of a low C3 on ESKD is broken down into components due to mediation alone, interaction alone, both mediation and interaction, and neither mediation nor interaction [27]).

## 3. Results

Of the 56 patients included in the study, 12 (21%) presented low serum C3 (LowC3) (normal range: 90–180 mg/dL) at the time of renal biopsy; clinical and laboratory parameters at renal biopsy are reported in Table 1.

The LowC3 group had by definition lower mean sC3 values (78.4 ± 10.9 vs. 117.6 ± 20.3 mg/dL, *p* < 0.000). However, mean sC4 values (23.8 ± 7.8 mg/dL vs. 28.5 ± 6.9 mg/dL; *p* = 0.048) were significantly lower too, although sC4 was within the normal range in the entire study cohort (normal range: 10–40 mg/dL). At biopsy, we did not observe significant differences in age, eGFR, 24-hour proteinuria, and laboratory parameters related to TMA (hemoglobin, platelets, and LDH).

The use of steroids did not differ significantly between the two groups, while the administration of RAASi was slightly more frequent in the non-LowC3 group. No patient had a history of resistant or malignant hypertension, and blood pressure was well controlled at the time of biopsy in all cases.

### 3.1. Histopathological Findings

We found substantial uniformity between the two groups in terms of MEST-C parameters and immunofluorescence positivity. We identified 17/56 (30%) patients with TMA, which coexisted with typical IgAN findings.

Of the 17 patients with TMA, 8 had acute TMA. The other nine had signs of chronic TMA. Two (4%) of the seventeen TMA patients had aHUS at disease onset. TMA was significantly more frequent in the LowC3 group [7/12 (58%) vs. 9/44 (20%), *p=* 0.02]. Considering other parameters such as age at onset, gender distribution, proteinuria, and eGFR, no significant differences were found between TMA-positive and TMA-negative patients. In addition, treatment approaches were similar in patients with and without TMA.

### 3.2. Renal Outcomes

The median follow-up was 4.6 years (range 0.5 month–11.0 years).

**Figure 1:**
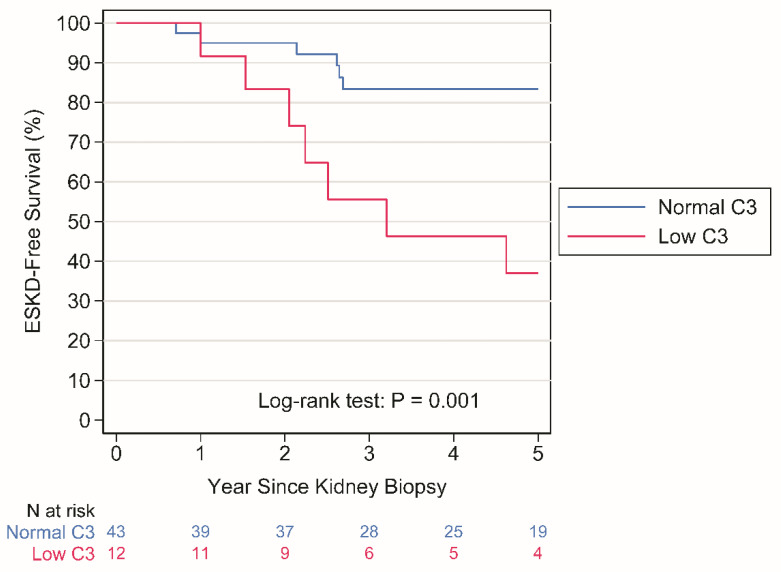
Kaplan–Meier plots of time to ESKD according to LowC3. ESKD, end-stage kidney disease.

Crude analysis. ESKD occurred in 8 patients with normal C3 levels over a period of 248 person years (py) (rate: 3.2 per 100 py) and in 8 patients with LowC3 levels over a period of 50 py (rate: 15.9 per 100 py), *p* = 0.001 by the log-rank test (Figure 1).

**Figure 2:**
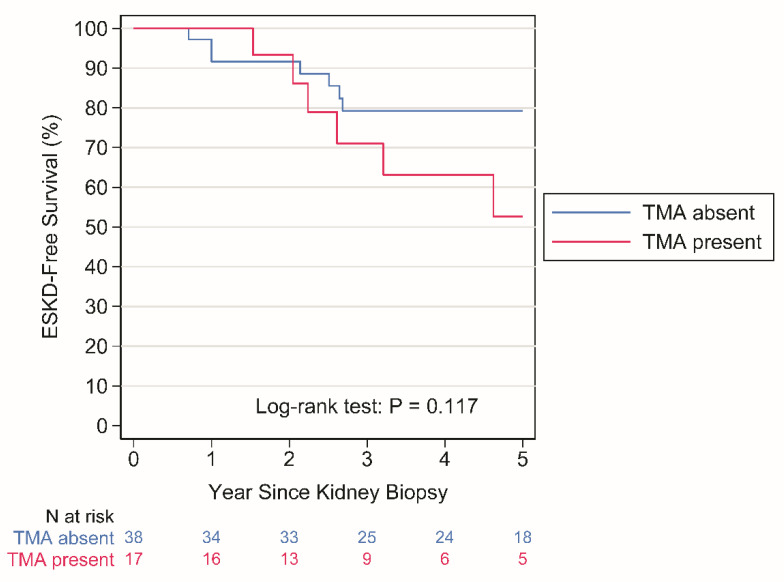
Kaplan–Meier plots of time to ESKD according to TMA at biopsy. ESKD, end-stage kidney disease; TMA, thrombotic microangiopathy.

ESKD occurred in 9 patients with no TMA over a period of 224 person years (py) (rate: 4.0 per 100 py) and in 7 patients with TMA over a period of 74 py (rate: 9.4 per 100 py), *p* = 0.117 by the log-rank test (Figure 2).

Multivariable-adjusted regression analysis. Results from crude and multivariable-adjusted Cox proportional hazards regression models are reported in Table 2. LowC3 was strongly associated with an increased hazard of ESKD (crude hazard ratio [HR]: 4.62 [95%CI: 1.71 to 12.48; *p* = 0.003] (Table 2).

The association was not affected after adjustment for sex, ethnicity, and use of RAASi (adjusted hazard ratio [aHR]: 5.84 [95%CI; 1.69 to 20.15; *p* = 0.005] (Table 2). In contrast, TMA was associated with a numerically increased hazard of ESKD, which was not statistically significant [HR: 2.17 [95%CI: 0.80 to 5.87; 0.126]; aHR: 2.21 [95%CI: 0.66 to 7.47; *p* = 0.200]] (Table 2). Most importantly, as shown in Table 2, when LowC3 and TMA were jointly included in the Cox regression model, the hazard ratio of LowC3 was virtually unaffected, whereas the point estimates of the hazard ratio of TMA became close to the null value of 1 (LowC3 HR, adjusted for TMA: 4.14 [95%CI: 1.47 to 11.68; *p* = 0.007]; LowC3 aHR, adjusted for TMA: 5.55 [95%CI: 1.46 to 21.01; *p* = 0.012]; TMA HR, adjusted for LowC3: 1.51 [95%CI: 0.53 to 4.30; *p* = 0.438], TMA aHR, adjusted for LowC3: 1.15 [95%CI: 0.30 to 4.48; *p* = 0.836].

#### Mediation Analysis

After adjustment for sex, ethnicity, and RAASi use, the estimated overall proportion of the relation between C3 and ESKD mediated by TMA was low and not statistically significant (overall proportion of the relation between LowC3 and ESKD mediated by TMA: 10% [95%CI: 0 to 62; *p* = 0.697]).

After additionally adjusting the LowC3+TMA Adj.+RAASi model for MEST-C score, which was not available in 6 patients, results were virtually identical (HR associated with LowC3: 6.09 [95%CI: 1.50 to 24.75; *p* = 0.012]; HR associated with TMA: 0.81 [95%CI: 0.19 to 3.46; *p* = 0.778]).

Moreover, the C3/C4 ratio did not predict renal prognosis better than LowC3 alone (Supplementary Tables S1 and S2).

## 4. Discussion

Albeit with the major limitation of a small sample size, which warrants further prospective and larger studies, our results provide evidence that C3 hypocomplementemia is associated with an increased risk of ESKD with mechanisms that are largely independent from TMA in a Northern Italian population. It additionally shows that the association, if any, between TMA and ESKD is entirely accounted for by C3 hypocomplementemia. This evidence might suggest that the overactivation of ACP is a key driver of disease progression in IgAN, whether it leads to TMA or not. Indeed, approximately 20% of patients in our study had low sC3 values at renal biopsy; these patients had a worse renal prognosis (Figure 1). Notably, in the LowC3 group, though sC4 was within normal limits, the latter was significantly lower and correlated strictly with sC3. Previous studies reported that about 20% of patients with IgAN had decreased sC3 levels at kidney biopsy [23,28], similarly to our findings; in a retrospective study [14], patients with decreased sC3 levels had more intense deposition of C3 in the mesangium than those who had normal sC3 levels, and serum C3 levels were significantly associated with progression to kidney failure. However, the predictive value of serum C3 was lower than clinical markers such as proteinuria and eGFR. In contrast, another study of 496 patients with IgAN, of whom 22% had low levels of C3, reported that the renal survival rate of the group with decreased C3 levels was lower than that of the group with normal C3 levels. However, upon propensity score matching, sC3 levels were not associated with disease progression [28]. Others have suggested that for IgAN, the C3/C4 ratio may be a better marker for disease activity and predictor of renal failure than sC3 levels alone [22]. Our study did not confirm this finding (Supplementary Materials). While the C3/C4 ratio is predictive in our cohort, low sC3 alone is more predictive than the C3/C4 ratio, possibly highlighting that the key driver of the association is C3 consumption per se.

In our study, on the other hand, the predictive role of ESKD of baseline C3 hypocomplementemia is prominent. As a result of not excluding patients with IgAN who had AKI or normal renal function, our study differed from that conducted by Yang X et al. [28].

Moreover, our study hints at the existence of a continuum between C3 hypocomplementemia and the appearance of TMA. The latter was significantly more frequent in the LowC3 cohort, particularly acute TMA. We hypothesize that the reduction in sC3 reflects a dysregulated activation of the ACP in IgAN, which could lead to the progression from “pure” IgAN to renal TMA, which in turn is responsible for severe hypertension [14].

We did not observe any differences between groups in terms of MEST-C scores. This might reflect the fact that there are no appreciable differences in terms of mesangial proliferation, focal segmental glomerulosclerosis, endocapillary hypercellularity, chronicity, or crescents between groups, or merely that our sample size is too small to identify any actual difference in the distribution of such lesions between patients with normal and low C3.

Previous reports [15,17] showed that renal prognosis was strictly related to acute but also chronic microangiopathic lesions at kidney biopsy and that endothelial damage until TMA was related to tubular atrophy, proteinuria, and hypertension. Based on our findings, it appears that TMA might be only an epiphenomenon of ACP overactivation and does not determine a worse renal prognosis by itself.

## 5. Conclusions

Our study has the major limitation of a small sample size, which warrants larger studies. However, low sC3 at the time of biopsy emerges as a clear predictor of CKD progression [29,30], strongly supporting the crucial role of complement overactivation/dysregulation as a driver of progression towards ESKD, further highlighting the rationale of ongoing pharmacological trials of complement blockade in IgAN [31].

## Figures and Tables

**Table 1:** Clinical and histopathological characteristics.

	Low Serum C3 at Biopsy		*p* Value
	No	Yes	
Number of pts (%)	44 (78.6)	12 (21.4)	
Age, years (*mean ± SD*)	46.7 ± 17.4	42.4 ± 17.5	0.496
Male (*n*, *%*)	33 (75)	6 (50)	0.072
Non-white ethnicity (*n*, *%*)	4 (9)	3 (25)	0.128
Hypertension, history	24 (45.5)	6 (50)	1.000
eGFR mL/min/1.73 m^2^ (*mean ± SD*)	54.2 ± 40.4	52.8 ± 46.5	0.775
Serum Albumin, g/dL (*mean ± SD*)	3.4 ± 0.6	3.2 ± 0.6	0.195
Proteinuria, g/day (*mean ± SD*)	2.6 ± 2.1	5.5 ± 7.7	0.383
C3, mg/dL (*mean ± SD*)	117.6 ± 20.3	78.4 ± 10.9	0.000
C4, mg/dL (*mean ± SD*)	28.5 ± 6.9	23.8 ± 7.8	0.027
Hb, g/dL (*mean ± SD*)	11.2 ± 2.3	12.4 ± 2.3	0.113
LDH, IU/L (*mean ± SD*)	397.0 ± 121.2	412.9 ± 103.2	0.450
Platelets (×1000/mm^3^) (*mean ± SD*)	216.4 ± 49.1	212.7 ± 63.3	0.829
Histology Immunofluorescence			
IF deposition—IgA (*mean ± SD*)	2.5 ± 0.6	2.6 ± 0.5	1.000
IF deposition—IgA (*mean ± SD*)	0.8 ± 0.5	1.0 ± 0.5	0.546
IF deposition—IgM (*mean ± SD*)	1.2 ± 0.6	1.4 ± 0.5	0.352
IF deposition—C3 (*mean ± SD*)	2.4 ± 0.6	2.4 ± 0.5	1.000
MEST-C (*n*, *%*)			
Mesangial	11 (25.0)	4 (33.3)	0.462
Endocapillary	11 (25.0)	3 (25.0)	0.720
Sclerosis	31 (70.4)	6 (50.0)	0.113
Tubular atrophy	23 (52.3)	6 (50.0)	1.000
Crescents	15 (34.1)	4 (33.0)	1.000
MEST-C score sum	2.4 (1.2)	2.3 (1.2)	0.667
TMA (*n*, %)	10 (22.7)	7 (58.3)	0.002
Acute	6	2	
Chronic	4	5	
Therapy (*n*, *%*)			
Prednisone	38 (86.3)	10 (83.3)	0.639
RAAS inhibitors	36 (81.8)	6 (50.0)	0.054
Other immunosuppressant	11 (25.0)	6 (50.0)	0.154

**Table 2:** Crude and multivariable adjusted cox proportional hazards multiple regression models for ESKD.

	LowC3-Crude	TMA-Crude	LowC3-Adj.	TMA-Adj.	LowC3-Adj.+RAASi	TMA-Adj.+RAASi	LowC3+TMA	LowC3+TMA-Adj.	LowC3+TMA Adj.+RAASi
LowC3	4.62 ***		7.98 ***		5.84 ***		4.14 ***	6.77 ***	5.55 **
	[1.71, 12.48]		[2.48, 25.76]		[1.69, 20.15]		[1.47, 11.68]	[1.90, 24.14]	[1.46, 21.01]
	0.003		0.001		0.005		0.007	0.003	0.012
TMA		2.17		3.00 *		2.21	1.51	1.46	1.15
		[0.80, 5.87]		[0.94, 9.59]		[0.66, 7.47]	[0.53, 4.30]	[0.42, 5.05]	[0.30, 4.48]
		0.126		0.064		0.200	0.438	0.552	0.836
Age, yrs			1.04 **	1.03	1.02	1.01		1.03 *	1.02
			[1.00, 1.07]	[0.99, 1.06]	[0.98, 1.06]	[0.97, 1.05]		[1.00, 1.07]	[0.98, 1.06]
			0.049	0.115	0.326	0.606		0.056	0.321
Non-white ethnicity			8.05 ***	6.97 ***	6.34 ***	5.23 ***		7.97 ***	6.42 ***
			[2.34, 27.66]	[2.13, 22.82]	[1.74, 23.14]	[1.55, 17.64]		[2.29, 27.70]	[1.74, 23.63]
			0.001	0.001	0.005	0.008		0.001	0.005
Male sex			1.24	1.27	1.27	1.19		1.33	1.31
			[0.42, 3.73]	[0.40, 3.99]	[0.41, 3.90]	[0.36, 3.90]		[0.43, 4.11]	[0.41, 4.16]
			0.697	0.688	0.674	0.776		0.621	0.648
RAASi					0.46	0.32 *			0.48
					[0.13, 1.66]	[0.10, 1.03]			[0.12, 1.87]
					0.236	0.057			0.291

Comparison of crude and multivariable adjusted hazard ratios of ESKD from Cox proportional hazard regression models that included LowC3, TMA, or both The table reports the hazard ratio of ESKD [95 percent confidence interval] and *p* value for each model; asterisks refer to the level of *p* value to ease the readability of the table as follows: <0.01 ***, <0.05 **, 0.1 *. ESKD, end-stage kidney disease; RAASi, renin angiotensin aldosterone inhibitors; TMA, thrombotic microangiopathy. LowC3-Crude, model including LowC3 only; TMA-Crude, model including TMA only; LowC3-Adj, model for LowC3 adjusted for age, ethnicity, and gender; TMA-Adj, model for TMA adjusted for age, ethnicity, and gender; LowC3-Adj.+RAASi, model including LowC3 additionally adjusted for RAASi; TMA-Adj.+RAASi, model including TMA additionally adjusted for RAASi; LowC3+TMA, model including both LowC3 and TMA; LowC3+TMA-Adj., model including both LowC3 and TMA adjusted for age, ethnicity, and gender; LowC3+TMA Adj.+RAASi, model including both LowC3 and TMA, additionally adjusted for RASSi.

## Data Availability

Because of the nature of this research, participants of this study did not agree for their data to be shared publicly, so supporting data are not available.

## Supplementary Materials

The following supporting information can be downloaded at https://www.mdpi.com/article/10.3390/jcm13092594/s1. Table S1: Crude and multivariable adjusted HR associated with the C3 to C3/C4 ratio from Cox PH regression models. Table S2: Statistical tests showing that the C3/C4 ratio is better than C3 alone to predict end-stage kidney disease.
