# Supplementary material for: C3 Hypocomplementemia Predicts the Progression of CKD towards End-Stage Kidney Disease in IgA Nephropathy, Irrespective of Histological Evidence of Thrombotic Microangiopathy
Source: J Clin Med. 2024 Apr 28;13(9):2594. doi: 10.3390/jcm13092594 (PMC11084933; doi:10.3390/jcm13092594)
Supplement: Supplementary file 1 [file jcm-13-02594-s001.zip › jcm-2886766-supplementary.pdf]

Table S1.Crude and multivariable adjusted HR associated with C3 to C4 ratio from Cox PH regression models

	**C3/C4-Crude**	**C3/C4-Adj.**	**C3/C4-Adj.+RAASi**	**C3/C4+TMA**	**C3/C4+TMA-Adj.**	**C3/C4+TMA Adj.+RAASi**
Point estimate	0.89***	0.91*	0.92	0.90**	0.92	0.92
[Lower bound of CI,Upper bound of CI]	[0.82,0.97]	[0.82,1.00]	[0.83,1.02]	[0.82,0.98]	[0.83,1.02]	[0.83,1.03]
p-value	0.009	0.051	0.103	0.018	0.108	0.153

Comparison of crude and multivariable adjusted hazard ratios of ESKD associated with C3/C4 ratio from Cox proportional hazard regression models

The table reports the hazard ratio of ESKD [95 percent confidence interval] and P value for each model; asterisks refer to the level of P value to ease the readability of the table as follows: <0.01 ***, <0.05 **, 0.1 *. ESKD, end stage kidney disease; RAASi, renin angiotensin aldosterone inhibitors ; TMA, thrombotic microangiopathy. LowC3-Crude, model including Low C3 only; TMA-Crude, model including TMA only; LowC3-Adj, model for Low C3 adjusted for age, ethnicity and gender; TMA-Adj, model for TMA adjusted for age, ethnicity and gender; LowC3-Adj.+RAASi, model including Low C3 additionally adjusted for RAASi; TMA-Adj.+RAASi, model including TMA additionally adjusted for RAASi; LowC3+TMA, model including both Low C3 and TMA; LowC3+TMA-Adj., model including both Low C3 and TMA adjusted for age, ethnicity and gender; LowC3+TMA Adj.+RAASi, model including both Low C3 and TMA, additionally adjusted for RASSi.

Table S2. Statistical tests that C3/C4 ratio is better than C3 alone to predict end stage kidney disease

C3/C4-Crude: Test that C3/C4 better than C3: P=0.079
C3/C4-Adj: Test that C3/C4 better than C3: P=0.215
C3/C4-Adj.+RAASi: Test that C3/C4 better than C3: P=0.279
C3/C4+TMA: Test that C3/C4 better than C3: P=0.096
C3/C4+TMA-Adj.: Test that C3/C4 better than C3: P=0.281
C3/C4+TMA Adj.+RAASi:Test that C3/C4 better than C3: P=0.317
